# Impact of antiplatelet therapy on microvascular thrombosis during ST-elevation myocardial infarction

**DOI:** 10.3389/fmolb.2024.1287553

**Published:** 2024-03-13

**Authors:** Sophia Khattak, Jonathan N. Townend, Mark R. Thomas

**Affiliations:** ^1^ Institute of Cardiovascular Sciences, University of Birmingham, Birmingham, United Kingdom; ^2^ Department of Cardiology, Queen Elizabeth Hospital, Birmingham, United Kingdom

**Keywords:** antiplatelet therapy, STEMI, coronary artery disease, inflammation, microvascular thrombosis

## Abstract

During an acute coronary syndrome, atherosclerotic plaque rupture triggers platelet activation and thrombus formation, which may completely occlude a coronary artery leading to ST-elevation myocardial infarction (STEMI). Although emergency percutaneous coronary intervention (PCI) is effective in re-opening the main coronary arteries, the downstream microvasculature can become obstructed by embolised plaque material and thrombus. Dual antiplatelet therapy is recommended by guidelines and used routinely for the management of STEMI to reduce the risk of recurrent atherothrombotic events. However it is unclear to what extent antiplatelet therapy reduces microvascular thrombosis, largely because most tools to assess microvascular thrombosis only became available after antiplatelet therapy was already used in the majority of patients. Platelets play a central role in key aspects of microvascular thrombosis, such as atherosclerotic plaque-induced thrombus development, inflammation and microvascular dysfunction, making them a potential target for novel therapeutic interventions. Currently, more potent antiplatelet agents like GPIIb/IIIa inhibitors may be administered during PCI directly into coronary arteries with high thrombus burden but it is not well-established whether this reduces microvascular thrombosis and they significantly increase the risk of bleeding. In this review article we discuss the role of platelets in microvascular thrombosis, describe how microvascular thrombosis and obstruction can be assessed clinically and explore potential new antiplatelet treatments for this. In particular, we highlight novel antiplatelet drugs targeting the platelet receptor GPVI, as well as PAR4, GPIb-IX-V and 5HT2A receptors. We also discuss the potential benefit of P-selectin inhibitors as they have proven to be effective in reducing microvascular thrombosis in sickle-cell disease which could translate into potential benefits in acute coronary syndrome.

## Introduction

During an acute coronary syndrome (ACS), thrombus formation within a coronary artery is initiated by atherosclerotic plaque rupture or erosion resulting in platelet activation by exposed subendothelial components. When thrombus completely occludes a coronary artery, the result is generally ST-elevation myocardial infarction (STEMI) which is characterised by ischaemia and rapid, extensive myocyte damage unless the thrombus is promptly dispersed, thereby restoring vessel patency. The most effective treatment is emergency percutaneous coronary intervention (PCI), which involves re-opening the blocked coronary artery with balloons and stents. The resulting rapid restoration of blood flow has been shown to reduce myocardial infarct size and preserve left ventricular (LV) systolic function more effectively than thrombolytic therapy (Bulluck et al., 2016). Antiplatelet therapy in addition to PCI is a cornerstone of treatment for these patients. Patients with STEMI usually receive immediate dual antiplatelet therapy consisting of aspirin and a platelet P2Y_12_ inhibitor, which is usually continued for 12 months to reduce the risk of recurrent atherothrombotic events. Furthermore, it also plays a vital role in preventing stent thrombosis.

Although mortality in acute STEMI has declined since the introduction of emergency PCI, it still has an in-hospital mortality rate of around 5%. Further mortality and morbidity occurs in approximately 8% of patients over the subsequent 12 months, the majority (over 50%) of which is due to a cardiovascular cause, such as recurrent ACS, left ventricular damage causing heart failure and ventricular arrhythmia (Santos et al., 2015). While this is in part due to late presentation preventing effective myocardial salvage despite PCI, there remains a significant risk of such events even in patients presenting within the “golden” early hours of symptoms. Much to the disappointment of clinicians, it was clear at an early stage of the development of emergency PCI, that despite apparent restoration of vessel patency, the restoration of myocardial blood flow was often poor. Only one-third of patients were found to have evidence of good myocardial blood flow as judged by ST segment resolution and myocardial blush (Cruz et al., 2022). It is thought that this is a result of coronary microvascular obstruction due to distal embolisation of thrombus as well as myocardial oedema. Furthermore, animal studies suggest that much myocardial injury occurs after restoration of blood flow due to reperfusion injury (Xia et al., 2016). It has been widely believed that effective antiplatelet therapy in patients with STEMI, given at the time of emergency PCI, would ameliorate distal embolisation and provide at least partial prevention of distal thrombus embolisation and microvascular obstruction.

In this article we provide an overview of the pathophysiology of microvascular thrombosis during STEMI, including its clinical assessment and the role of platelets and antiplatelet therapy, including potential novel therapies which show promising results at early stages of development.

## Role of platelets in the pathophysiology of coronary obstruction in STEMI

**FIGURE 1:**
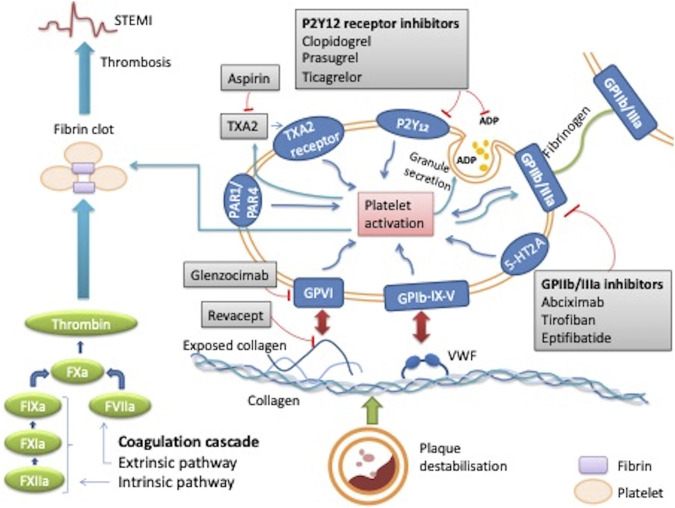
Mechanism of action of platelets in thrombus formation during STEMI and the effect of antiplatelet therapy. When plaque destabilisation occurs, due to plaque rupture or erosion, initial platelet tethering is mediated by the interaction between the complex glycoprotein (GP) Ib-IX-V and Von Willebrand factor (VWF). Various intracellular signalling reactions occur resulting in inside out activation of key integrin receptors such as GP IIb/IIIa and GP1a/IIa causing stable platelet adhesion. This stimulates local mediators such as ADP, thomboxane A2 and thrombin thus amplifying platelet activation and ultimately leading to thrombus formation. Sites of action of common antiplatelet agents are reported: aspirin inhibits thromboxane TXA2; clopidogrel, prasugrel and ticagrelor are inhibitors of the ADP P2Y12 receptor, abciximab, eptifibatide, tirofiban are GPIIb/IIIa receptor inhibitors. Revacept and glenzocimab are inhibitors of the collagen-induced platelets activation, which is mediated by GPVI. ADP: adenosine diphosphate; GP: glycoprotein; PAR-1: platelet protease-activated receptor-1; TXA2: thromboxane A_2_; VWF: von Willebrand factor; 5HT_2A_: serotonin receptor.

Platelets play a key role in the pathogenesis of STEMI. Atherosclerotic plaque rupture or erosion causes the formation of a platelet-rich thrombus. At the site of the vascular injury, the damaged vessel wall exposes platelet agonists that activate platelets by interacting with their surface receptors. This formation of platelet-rich thrombus is complex and involves multiple phases of platelet activity such as tethering, rolling, adhesion, activation, spreading, granule release and aggregation (Alenazy and Thomas, 2021) (Figure 1).

Platelet adhesion is initiated by exposed sub-endothelial components of the damaged wall such as collagen and Von Willebrand factor (VWF). The exposed collagen attaches to the GPVI and integrin α2β1, which are the main receptors for collagen on platelets. GPVI induces platelet activation and release of secondary mediators such as adenosine diphosphate (ADP), thromboxane A_2_ and 5-HT2A within platelets to further amplify platelet activation. The release of mediators contribute to platelet aggregation. This is mediated by inside-out activation which converts inactive GP IIb/IIIa on the surface membrane into the active form. Activated GPIIb/IIIa then binds fibrinogen, which bridges the platelets together. Many other factors also result in further platelet activation, including activation of the coagulation cascade and subsequent production of thrombin, which may also activate protease-activated receptor (PAR)-1 and PAR-4 on the platelet surface. These protease receptors cause further potent activation, aggregation, granule release and shape change of the platelets (Alenazy and Thomas, 2021) (Figure 1).

Platelets serve both thrombotic and inflammatory functions. Platelets play a role in inflammation by interacting with leukocytes (particularly neutrophils and monocytes) and forming platelet-leukocyte aggregates, amplifying pro-inflammatory responses and influencing various immune and repair processes (Thomas and Storey, 2015; Lisman, 2018). The mode of interaction is in part by direct binding of receptors on platelets with ligands on leukocytes, such as P-selectin on the platelet surface interacting with P-Selectin Glycoprotein Ligand 1 (PSGL1) on leukocytes and GP1b∝ on the platelet surface interacting with Mac-1 on leukocytes. Furthermore, these interactions enhance leukocyte activation and release of pro-inflammatory mediators such as elastase, cathepsin G (Stark and Massberg, 2021) and S100A8/A9 (Lisman, 2018; Colicchia et al., 2022). The downstream effect of these platelet-leukocyte interactions also results in increased leucocyte phagocytic activity, increased production of reactive oxygen species, increased transmigration of leukocytes over the endothelial lining, production of leukotrienes, activation of coagulation via tissue factor, initiation of tissue repair and generation of neutrophil extracellular traps (NETs) (Lisman, 2018).

Circulating leukocytes also play a central role in atherothrombosis. Patients with STEMI are found to have a high volume of circulating activated polymorphonuclear cells (neutrophils, eosinophils and basophils) and high levels of neutrophils in particular are associated with poor outcomes (Thomas et al., 2021). Furthermore, platelet-neutrophil interactions are also key to the release of NETs, which are essentially chromatin consisting of DNA segments in sequence around 8 histone protein (and other granule protein) cores. NETs have pro-inflammatory, cytotoxic and prothrombotic effects and also form part of the scaffold of the thrombus in STEMI (Döring et al., 2020). The release of NETs triggered by platelet-neutrophil interactions in STEMI contributes to microvascular obstruction through physical occlusion, thrombotic formation, and inflammatory effects thus worsening tissue perfusion and causing myocardial death. Coronary NET burden has also been shown to have a positive correlation with infarct size in patients with STEMI also suggesting that NETs could be playing a significant role in microvascular obstruction and contributing to myocardial damage (Mangold et al., 2015).

Platelets play a crucial role in STEMI by forming platelet-rich thrombi when atherosclerotic plaques rupture. The interaction of platelets with surface receptors and release of immune and inflammatory response, results in microvascular obstruction causing myocardial death.

### Coronary microvascular dysfunction and obstruction

Even after restoring flow in the epicardial coronary arteries, flow in the microvasculature is not restored in approximately 50% of cases resulting in increased infarct size and reduced left ventricular (LV) function, thus leading to unfavourable long-term outcomes (Marc et al., 2019). During STEMI, plaque rupture or erosion can result in distal embolisation of material (Topol and Yadav, 2000). In addition, during PCI, manipulation of the plaque with guidewires, ballooning and stenting can also result in embolisation downstream (Niccoli et al., 2016). This may physically block myocardial perfusion depending on the volume and size of the embolised plaque material but may also result in microvascular obstruction by release of inflammatory mediators and vasoactive substances causing myocardial oedema and vasoconstriction. Finally, endothelial cell necrosis can occur within the capillary bed contributing further to the obstruction (Wu et al., 1998).

There are a number of other factors that also influence the development of coronary microvascular obstruction. These include comorbidities, extent of ischaemic injury and severity of interstitial myocardial oedema and reperfusion injury (Niccoli et al., 2016). Comorbidities that predispose towards microvascular obstruction include diabetes mellitus, hypercholesterolaemia, acute hyperglycaemia or pre-existing coronary microvascular dysfunction (Crea et al., 2014). The severity of ischaemic injury also depends on the duration and extent of ischaemia, which is affected by how long it takes to re-open the coronary artery and the size of the coronary territory involved (Niccoli et al., 2016). Ischaemic myocardial injury initiates an acute inflammatory response resulting in myocardial damage by release of oxygen-derived free radicals, proteases and leukotrienes (Thomas and Storey, 2015).

The association of inflammation with acute myocardial infarction has been recognised for over 50 years and it is also increasingly recognised that reperfusion injury has a major role in the pathophysiology of myocardial infarction. Reperfusion stimulates the production of reactive oxygen species by the mitochondria and other mediators of inflammation causing both tissue injury and myocardial oedema. Interstitial myocardial oedema plays an important role in further decreasing the flow through these damaged vessels by compressing the capillaries and small arterioles. Mitochondrial swelling and cell rupture can also cause an increase in the infarct size (Niccoli et al., 2016). Reperfusion injury is also exacerbated by obliteration of the vessel lumen by neutrophil-platelet complex that release vasoconstrictors and inflammatory mediators. Reperfusion injury may cause intramyocardial haemorrhage (IMH) which is associated with adverse LV remodelling and poor prognosis. IMH occurs by erythrocyte extravasation through severely damaged endothelial walls of the coronary arteries. It is associated with large infarct size, poor LV function and a lack of improvement at follow up. It is prevalent in nearly half of successfully revascularized acute myocardial infarctions (Calvieri et al., 2015).

In STEMI, microvascular obstruction is a problem, leading to increased infarct size and reduced LV function. Factors like plaque embolisation, inflammation-induced vasoconstriction, ischaemia duration as well as comorbidities contribute to microvascular obstruction. Reperfusion injury worsens outcomes with tissue damage, oedema and intramyocardial haemorrhage.

### Treatment of coronary microvascular obstruction

**FIGURE 2:**
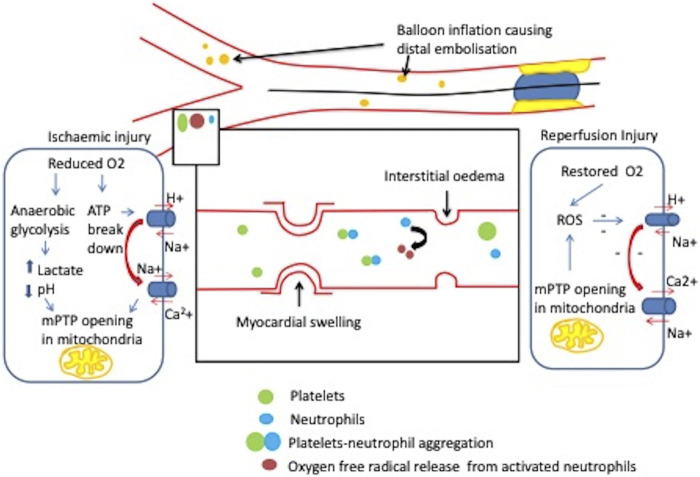
Coronary microvascular obstruction during STEMI. Balloon inflation at the site of plaque rupture causes downstream distal embolisation causing mechanical obstruction as well as a cascade of events such as activation of platelets and release of inflammatory mediators. Lack of coronary perfusion results in ischaemic injury during MI and starts ATP hydrolysis which increases intracellular Na+ and Ca 2+. This with raised lactate and decreased pH increases mitochondrial permeability transition pore opening (mPTP). When the patency of the vessel is preserved, during reperfusion state there is an increased mPTP opening which elevates reactive oxygen species (ROS) generation and disrupts intracellular distribution of Ca2+, Na+ and pH resulting in irreversible cell death (Xia et al., 2016).

Although some of these factors are non-modifiable such as genetic predisposition and pre-existing microvascular disease, the extent of reperfusion injury can be minimised by reduction in the time to re-open the blocked coronaries (improved with reduction in door to balloon time), although other factors also play a part such as Thrombolysis in Myocardial Infarction (TIMI) flow in the infarcted artery and whether there is an established collateral blood supply. Effective treatment of myocardial reperfusion injury is still lacking however. Although a number of agents have been examined in animal models with apparent success, to date no drug has been shown to reduce infarct size in humans after STEMI. This may be in part because in most cases, infarct size is determined by three factors: the extent of initial injury due to vessel occlusion, a time dependent process; myocyte reperfusion injury caused by free radicals and mediators of inflammation and microvascular obstruction causing ongoing ischemic injury. Early detection with coronary angiography and cardiac magnetic resonance imaging as well as management of microvascular obstruction with early reperfusion and pharmacological intervention probably represents a more treatable target than the complex problem of reperfusion injury and has the potential to improve morbidity and mortality in these patients (Niccoli et al., 2016; Alenazy and Thomas, 2021) (Table 1; Figure 2).

**TABLE 1:** Pathogenesis of coronary microvascular obstruction (MVO).

1a	Genetic predisposition and comorbidities	Ischaemic pre-conditioning can be a protective factor against MVO.
		Genetic factors that increase susceptibility to MVO include 1976T.C polyporphism of the adenosine 2A, genetic variations within defined regions of VEGFA and CDKN2B-AS1 genes, sex-specific allelic variants within MYH15, VEGFA and NT5E genes (Niccoli et al., 2016)
1b	Pre-existing coronary microvascular obstruction	Impaired coronary flow reserve can occur in patients with increased age and certain health conditions such as hypertension, diabetes, dyslipidaemia, insulin resistance and chronic inflammatory diseases (Crea et al., 2014)
2	Ischaemic Injury	Important clinical predictors of MVO are the ischaemia duration and extent confirmed on ECG by ST resolution, echocardiogram, CMR and invasive coronary indices to measure MVO (Niccoli et al., 2016)
3	Reperfusion Injury	Generally occurs when ischaemia lasts >3 h. MVO is caused by neutrophil-platelet aggregates that produce vasoconstrictors and inflammatory mediators, obliterating the vessel lumen (Thomas and Storey, 2015)
4	Distal Embolisation	Re-opening of blocked coronary arteries can cause distal embolisation of plaque material. The embolised material is thought to be biologically active and aggravates reperfusion injury even if the microcirculation is not mechanically obstructed. Myocardial perfusion generally falls when these embolised particles obstruct >50% of the coronary capillaries (Topol and Yadav, 2000)

## Clinical assessment of microvascular thrombosis during STEMI

With the help of invasive and non-invasive modalities, it is possible to detect microvascular obstruction (MVO) caused by microvascular thrombosis in patients after STEMI (Kumar et al., 2011) (Table 2).

**TABLE 2:** Clinical assessment of microvascular obstruction (Giugliano et al., 2004; Bulluck et al., 2017).

Non-invasive parameters	Thrombolysis in myocardial infarction (TIMI) flow	TIMI is a widely used visual assessment of flow appearance that provides immediate evaluation of microvascular reperfusion. It ranges from TIMI 0–3 with TIMI 3 being complete antegrade flow following intervention
		Angiographic definition of MVO is TIMI grade <2 (Bulluck et al., 2017)
	ECG ST segment resolution	ST-segment resolution of <70% at 60–90 min post reperfusion in the presence of a patent epicardial coronary artery is suggestive of MVO (Schröder, 2004)
	Myocardial blush	Grade 0: Absence of myocardial blush, contrast density or persistent “staining” suggestive of leakage of the contrast medium into the extracellular space
		Grade 1: Minimal myocardial blush or contrast density
		Grade 2: Moderate myocardial blush or contrast density but less than that obtained from the contralateral or ipsilateral non-infarct related coronary artery
		Grade 3: Normal myocardial blush or contrast density, comparable with that obtained during angiography of a contralateral or ipsilateral non-infarct related artery. (Van 't Hof et al., 1998) MBG of 0–1 is associated with increased all-cause mortality in patients with STEMI and a score or 2–3 is strongly associated with reduced all-cause mortality (Rehman et al., 2021; Cruz et al., 2022).
	Cardiac Magnetic Resonance	MVO is detected with early gadolinium enhancement by presenting as low signals or hypo-enhacement (dark areas) on CMR. These dark zones of no-reflow tend to fill in (appear bright) on delayed sequences due to penetration of gadolinium into the damaged capillaries.These dark regions correlate with no reflow regions suggesting MVO (Marcus et al., 1990; Wu et al., 1998).
Invasive parameters	Flow Based Parameters	CFVR is the ratio of hyperaemic coronary flow: resting flow measured using a Doppler velocity wire. It reflects epicardial and microvascular vasodilator capacity. CFR ratio of ≥2 is considered normal (Van De Hoef et al., 2013; Bulluck et al., 2016)
		Deceleration time of diastolic coronary flow velocity: Rapid deceleration time <600 m is associated with MVO and poor long term outcomes after reperfusion (Hirsch et al., 2008).
		Systolic flow reversal: The presence of early systolic flow reversal in intracoronary Doppler recordings is associated with MVO (Furber et al., 2004).
	Resistance Based Parameters	IMR is defined as distal coronary pressure times the mean transit time of a 3 mL bolus of saline at room temperature during maximal coronary hyperaemia. It is a thermodilution-derived index, measured using a guidewire that combines a pressure and temperature sensor.
		IMR <25 U is indicative of normal microvascular perfusion (Furber et al., 2004).
		HMR is the ratio between hyperaemic mean distal pressure and hyperaemic average Doppler flow peak velocity. HMR ≥2.5 suggests MVO (Berry et al., 2015).
		Pzf is defined as the distal coronary pressure when theoretically there is no flow in the coronary artery (Patel et al., 2015).

### Myocardial blush grade (MBG)

MBG was first described in 1998 (Van 't Hof et al., 1998) and is a method to assess myocardial perfusion in the region of the infarct-related coronary artery immediately after primary coronary angioplasty. It assesses the intensity of tissue radiopacity obtained by injecting contrast medium into the epicardial coronary arteries and how quickly the contrast dissipates. It is graded semi-quantitatively from 0 to 3 and explained in further detail in Table 2.

### Cardiac magnetic resonance assessment of MVO

Cardiac Magnetic Resonance (CMR) is commonly used to measure infarct size as well LV function and viability of LV segments in STEMI patients. MVO in patients with STEMI detected on CMR is associated with nearly four-fold increased risk of major adverse cardiovascular events (MACE) in the 2 years after MI, compared to when MVO is absent (Van Kranenburg et al., 2014). The patient prognosis relates to the extent of the myocardial injury during coronary occlusion (Eitel et al., 2014; Van Kranenburg et al., 2014) (Table 2).

### Invasive measurement of coronary microcirculation

Invasive assessment of the coronary microcirculation at the time of angiography and PCI, allows the early detection of microvascular obstruction which has the potential to direct treatment. It can be broadly be divided into flow- and resistance-based parameters (Marcus et al., 1990).

### Flow-based parameters

These can be measured using Doppler and thermodilution techniques and include the coronary flow reserve (CFR), deceleration time of diastolic coronary flow velocity and the presence of systolic flow reversal. A CFR ratio of ≥2 is normal and a value of <2 has been shown to correspond with extent of MVO by CMR and is associated with increased mortality at 10 years (Van De Hoef et al., 2013). A CFR value of <2 has a sensitivity of 79% in detecting MVO and 80% in detecting intramyocardial haemorrhage (IMH) on CMR. However, the test has a low specificity of 34% (Marc et al., 2019). After successful emergency PCI, rapid deceleration time of coronary diastolic flow velocity (<600 m) and the presence of early systolic flow reversal assessed by intracoronary Doppler are associated with a larger extent of MVO and poor long-term outcomes after reperfusion (Iwakura et al., 1996; Furber et al., 2004; Hirsch et al., 2008).

### Resistance-based parameters

After successful reperfusion following emergency PCI, there is increased microvascular resistance in the coronary arteries and invasive indices of microvascular resistance may be better than CFR in predicting all cause death or heart failure (Carrick et al., 2016a; Carrick et al., 2016b). These include the index of microvascular resistance (IMR), hyperaemic microvascular resistance (HMR) and coronary zero-flow pressure (Pzf).

### Index of microvascular resistance

IMR is an important surrogate parameter that allows for quantification of MVO and has been used to assess coronary microcirculation in STEMI patients (Fearon et al., 2003). A guidewire with a pressure and temperature sensor is placed in the distal third of the vessel and an average of 3 transit times of 3 mL of room temperature normal saline during peak hyperaemia are used to calculate mean transit time. An IMR value of <25 indicates normal microvascular perfusion (Pijls et al., 2002; Berry et al., 2015). Higher IMR has been shown to correlate with a higher likelihood of MVO, a larger MI size and worse LV function at follow up by echocardiography and CMR (McGeoch et al., 2010; Carrick et al., 2016b; Bulluck et al., 2016). Patients with IMR >40 measured immediately after emergency PCI were more likely to have MVO on CMR, associated with worse LV remodelling by CMR at 6 months and an independent predictor of mortality or heart failure (Carrick et al., 2016a; Bulluck et al., 2016). IMR is an invasive marker with associated risks and it is therefore not routinely performed after the index procedure unless there is a need for further intervention. Furthermore, manual injections of normal saline are prone to inter- and intra-observer variability adding to some variability in the measurements obtained.

### Hyperaemic micovascular resistance and coronary zero-flow pressure

Hyperaemic microvascular resistance (HMR) and coronary zero-flow pressure (Pzf) are measured simultaneously using a coronary guide wire with a doppler transducer and pressure sensor (Kitabata et al., 2009). In patients with STEMI, HMR has been less studied than IMR, however, small studies have shown HMR to be indicative of MVO by CMR and reduced myocardial flow on Positron emission tomography (PET) (Ahn et al., 2016). Pzf is distal coronary pressure when theoretically the flow in the distal coronary artery approaches zero. This is not possible to measure directly as in real life the flow in the distal coronary artery would never cease under normal circumstances and so Pzf is extrapolated from pressure velocity loops. Pzf is not currently widely available and is only a research tool (Patel et al., 2015).

One or more of these tests may be used in the detection of MVO in patients with STEMI. This would allow early identification of these high-risk patients and potentially immediate treatment.

### Overview of the use of antiplatelet therapy in STEMI

Current guidelines for STEMI support immediate treatment with antiplatelet therapy and the effects of antiplatelet therapy on microvascular thrombosis are incompletely understood. Current treatment includes aspirin, and a P2Y_12_ inhibitor, such as prasugrel or ticagrelor. Since the antithrombotic benefit of dual antiplatelet therapy (DAPT) generally outweighs the risk of bleeding for at least the first year after STEMI, the European Society of Cardiology (ESC) and American College of Cardiology/American Heart Association (ACC/AHA) guidelines recommend DAPT for 12 months in patients with low bleeding risk and 6 months in those with a higher risk of haemorrhage (Valgimigli et al., 2018).

There are many distinct pathways of platelet activation and aspirin and a P2Y_12_ inhibitor may not be sufficient to block all of these. This may in part explain why some patients with ACS experience recurrent thrombotic events even if they are treated with DAPT for up to a year. More potent antiplatelet agents such as the glycoprotein (GP) IIb/IIIa inhibitors are available for use intravenously during PCI. Evidence of benefit for routine use of these drugs in the modern era exists but is modest and currently their use is largely restricted to patients with a heavy thrombus burden: an approach largely lacking in supporting evidence. A major concern around the use of GPIIb/IIIa inhibitors is their severe impact on haemostasis, resulting in an increased risk of bleeding, and they can also cause thrombocytopenia (Zheng et al., 2020). Further research is needed to identify new treatment strategies such as development of new antiplatelet drugs that target platelet receptors other than P2Y_12_ and GPIIb/IIIa receptors that might reduce microvascular obstruction and infarct without increasing bleeding risk.

### Current and novel antiplatelet medications

Although DAPT inhibits the P2Y_12_ receptor and thromboxane A_2_ (TXA_2_) synthesis, there are still multiple platelet activation mechanisms and pathways that are minimally affected, thus still allowing platelet activation and aggregation that leads to atherothrombotic events. At 1 year after ACS, MACE occurs in around 10% of patients, death from recurrent MI occurs in around 6%–7% and stent thrombosis in <2% of the patients (James et al., 2012).

### Aspirin- cyclooxygenase (COX) enzyme inhibitor

Aspirin was the first antiplatelet drug shown to have a proven benefit in coronary artery disease (Cairns et al., 1985). Current guidelines recommend a loading dose of aspirin (300 mg) for the treatment of ACS in all patients followed by a maintenance dose (75 mg), unless there is a contraindication. Dual antiplatelet therapy (including aspirin) is routinely prescribed for 1 year, followed by aspirin monotherapy lifelong in most patients (Layne and Ferro, 2017).

Low-dose aspirin is predominantly an inhibitor of cyclo-oxygenase (COX)-1. COX-1 mediates the synthesis of TXA_2_ which is produced following platelet activation. This amplifies platelet activation and aggregation by activating thromboxane receptors. Inhibition of COX-1 by aspirin therefore reduces platelet activation and aggregation (Layne and Ferro, 2017). Data from the ISIS-2 trial showed unequivocally that aspirin reduces mortality in patients with STEMI but these patients were treated with thrombolysis rather than emergency PCI. It is unlikely that any trial will ever re-examine the efficacy of aspirin in this setting although the differences between the two modes of restoration of vessel patency are considerable (Calvieri et al., 2022). Few studies have looked at dose responses of aspirin in STEMI. One study showed that aspirin loading with 500 mg, compared to 250 mg may further reduce MVO, infarct size and adverse remodelling as assessed by CMR (Calvieri et al., 2022). Furthermore, impaired microcirculatory perfusion after PCI may in part be related to levels of thromboxane B_2_, which is inhibited by aspirin (Basili et al., 2014).

### P2Y_12_ inhibitors

In addition to aspirin, current guidelines mandate that patients with STEMI also receive a second antiplatelet agent such as prasugrel or ticagrelor; the less potent agent clopidogrel is not a first line agent. These are all ADP receptor antagonists that bind selectively to P2Y_12_ receptors to inhibit platelet function (Layne and Ferro, 2017).

Although there is evidence of improved morbidity and mortality in ACS patients with the use of P2Y_12_ inhibitors when used in addition to aspirin on a macrovascular level (Thomas and Storey, 2016), little evidence is available of the benefits at a microvascular level. Willowby et al. showed an improved cardiovascular function with clopidogrel with an increase in reactive hyperaemic index when compared to placebo in patients with stable coronary artery disease (Willoughby et al., 2014).

Ticagrelor is a nonthienopyridine antiplatelet which reversibly binds to ADP P2Y_12_ receptor. It has shown to provide a more potent inhibition of platelet activity compared to clopidogrel with reduced ischaemic events and mortality in patients with ACS (Wallentin et al., 2009). Ticagrelor has also shown to reduce microvascular injury in patients with ACS compared to clopidogrel with or without ST elevation MI (Park et al., 2019; 2016). Another study comparing ticagrelor with clopidogrel has also shown improved myocardial perfusion and left ventricular function in patients, thus reducing major adverse cardiovascular events for patients with ST elevation MI undergoing emergency PCI. Although the exact mechanism for this is not known, it is suggested that this beneficial effect on coronary microvascular dysfunction is likely from inhibition of cellular uptake of adenosine (Wang et al., 2019).

P2Y_12_ inhibitors are administered orally and it can take several hours to achieve their antiplatelet effect in the settings of a STEMI. Ubaid et al. compared initial cangrelor (an intravenous P2Y_12_ inhibitor with an onset of action of 1–3 min) infused for up to 2 h followed by ticagrelor to oral ticagrelor given at the time of the PCI procedure in combined invasive and CMR study of microvascular function and infarct size. Although more potent P2Y_12_ inhibition was observed in the cangrelor group, microcirculatory function and infarct size were not different within the groups (Ubaid et al., 2019). This important study suggests that either platelet function is not a critical determining factor in the causation of MVO (contrary to most current opinion) or that the platelet mechanism that is involved is not mediated by P2Y_12_.

### GP IIb/IIIa inhibitors

GP IIb/IIIa is a platelet integrin that mediates formation of bridges between platelets via binding to fibrinogen, which cross-links GPIIb/IIIa on nearby platelets, thereby causing platelet aggregation. GPIIb/IIIa inhibitors are thought to possibly improve vessel patency after PCI and recent studies have also suggested improved myocardial perfusion by inhibition of the interaction of platelets with the microvasculature (Layne and Ferro, 2017). GP IIb/IIIa inhibitors such as tirofiban, abciximab and eptifibatide are therefore currently used in clinical practice in selected acutely infarcted patients after coronary arteries are visualised on angiography, largely as a “bailout” or rescue treatment if there is a heavy thrombus burden or thrombotic complication as per operator discretion (Layne and Ferro, 2017) but this is based on expert opinion rather than evidence from clinical trials (Collet et al., 2021). GPIIb/IIIa inhibitors are usually given intravenously although there is some evidence that intracoronary administration of abciximab may be superior to intravenous treatment in improving myocardial perfusion, infarct size and MVO as shown by CMR (Thiele et al., 2008). Meta-analysis of studies including over 8,000 patients and a large UK analysis of over 100,000 patients has shown that adjunctive therapy with GP IIb/IIIa resulted in reduced MACE, 6–12 months mortality and the reinfarction rate although these trials largely pre-dated the introduction of prasugrel and ticagrelor which likely make some of the benefit of GPIIb/IIIa inhibitors redundant (Karathanos et al., 2019; Orzalkiewicz et al., 2019).

There is also some evidence that GP IIb/IIIa may improve LV function and microvascular integrity (Layne and Ferro, 2017). Rates of ischaemia and subacute stent thrombosis in stenting for myocardial infarction were reduced early by abciximab during the first several weeks but it did not appear to provide additional protection from late re-occlusion, late cardiac events or angiographic restenosis (Stone et al., 2002). Improved peri-procedural epicardial coronary artery flow has also been observed in STEMI patients treated with abciximab. An improvement in LV function was also noted in the abciximab group suggesting a possible effect on MVO. This study, which was mainly conducted via femoral access, did however note a higher incidence of groin haematomas in the abciximab group (Montalescot et al., 2001). This increased bleeding rate has been a consistent feature of GP IIb/IIIa usage and has been largely responsible for the steep decline in routine use of these drugs over the last decade (Le May et al., 2009; Collet et al., 2021).

Currently, GPIIb/IIIa inhibitors are used in selected patients with STEMI at the operator’s discretion, usually when there is a high thrombotic burden identified on coronary angiography. This restricted use is mainly because of a high bleeding risk and occasional severe thrombocytopaenia associated with their use together with increasing confidence in the use of modern potent oral P2Y_12_ drugs (Le May et al., 2009; Layne and Ferro, 2017; Zheng et al., 2020).

### GPVI inhibitors

The GPVI receptor plays a central role in platelet activation induced by atherosclerotic plaque rupture, a complex process involving multiple platelet receptor-ligand interactions (Alenazy et al., 2023). GPVI interacts with exposed subendothelial collagen, which induces intracellular signalling and platelet activation and aggregation. Release of secondary mediators, such as ADP and thromboxane A_2_ amplifies the response leading to sustained activation and aggregation. Furthermore, interaction of GPVI with polymerised fibrin, which is formed by the coagulation cascade, also activates platelets thereby supporting thrombus growth and thrombus stabilisation (Mammadova-Bach et al., 2015; Alenazy and Thomas, 2021). Platelet and leukocyte interaction leads to recruitment and activation of inflammatory cells and this is thought to mediate ischaemic inflammation and reperfusion injury increasing infarct size (Pachel et al., 2016). GPVI inhibition reduces the neutrophils within the infarcted zone and irreversibly downregulates circulating platelets by membrane shedding, which is an important mechanism in cell adhesion and inflammation (Mammadova-Bach et al., 2015; Pachel et al., 2016). Inhibition of GPVI has shown beneficial antithrombotic effects, a reduction in the infarct size and improved microvascular reperfusion in animal model treated with anti GPVI compared with anti-GPIb and anti-GPIIb/IIIa antibodies (Pachel et al., 2016). GPVI is seen as a promising target for novel antiplatelet treatment which would be likely to have potent antiplatelet effects with little increase in the risk of bleeding because it is believed that GPVI is not required for physiological haemostasis (Alenazy and Thomas, 2021). This is supported by the finding that genetic or acquired GPVI deficiency is manifest as a mild bleeding disorder only. Similarly genetic depletion of GVI in animal studies is thought to have little effect on haemostasis (Boulaftali et al., 2018).

The immune system plays an important role in reperfusion injury post MI. Neutrophil infiltration within minutes of cardiac injury and platelet-neutrophil interactions are thought to cause capillary plugging contributing to the no-reflow phenomenon and increasing infarct size. It is suggested that GPVI plays an important role in inflammation in addition to its thrombotic effects. An animal study of the Fc receptor γ chain, which are present in the platelet membrane and essential for expression and signal transduction of GPVI receptor found that mice deficient in this receptor were protected from myocardial ischaemia reperfusion injury. These mice also had reduced myeloperoxidase activity, which is an indicator of neutrophil activation (Takaya et al., 2005). A study on mice using the GPVI antibody JAQ1 as an inhibitor of the receptor found reduced neutrophil numbers in the infarct zone and smaller infarct size. There was also noticeable improvement in microperfusion in the ischaemic area after reperfusion (Pachel et al., 2016). It has not been possible to find a small molecule inhibitor of GPVI, but novel antagonists have now been produced (described in the following paragraphs) and it is also possible to block GPVI-mediated platelet responses by inhibition of Btk and Syk which are part of its downstream signalling pathway (Harbi et al., 2022; Harbi et al., 2021; Smith et al., 2022).

### Revacept

Revacept is a recombinant GPVI-Fc complex and a competitive GPVI inhibitor. It blocks platelet activation by binding to exposed collagen at the site of vascular injury, thereby preventing the exposed collagen from interacting with GPVI on platelets (Alenazy and Thomas, 2021). There is pre-clinical evidence from animal models that revacept decreases infarct size, reduces progression of atherosclerosis, and maintains cardiac perfusion (Schönberger et al., 2012). It also reduces platelet degranulation and the release of pro inflammatory cytokines thus playing a part in the anti-inflammatory process in the infarcted arteries. Furthermore, in these studies revacept did not cause thrombocytopenia, prolongation of bleeding time or any other adverse effects (Massberg et al., 2004; Schönberger et al., 2012). Revacept has been tested in humans in a phase 2 trial in which a high dose of revacept in addition to DAPT showed an increased platelet inhibition without an association with higher bleeding. The study did not reduce the primary end point which was the reduction of myocardial injury in patients with stable ischaemic heart disease undergoing percutaneous coronary intervention (Mayer et al., 2021).

### Glenzocimab

Glenzocimab (formerly known as ACT017) is a humanised Fab fragment of a monoclonal antibody which reversibly inhibits GPVI-mediated platelet aggregation induced by collagen (Alenazy and Thomas, 2021). Glenzocimab is derived from Fab9012, a mouse monoclonal antibody specific for GPVI (Voors-Pette et al., 2019). It is suggested that in addition to its antithrombotic effects, glenzocimab could also be useful in patients with ongoing thrombosis as it also limits thrombus growth once thrombus has already been formed by preventing GPVI/fibrinogen mediated platelet aggregation and activation (Ahmed et al., 2020). Glenzocimab has been tested in a phase 1 trial demonstrating safety and tolerability (Voors-Pette et al., 2019) as well as in acute ischaemic stroke patients in the ACTIMIS study. Glenzocimab was administered to patients with ischaemic stroke treated with recombinant tissue plasminogen activator with or without thrombectomy. Compared to placebo, glenzocimab reportedly caused a nominally lower incidence of intracerebral haemorrhage and death and a favourable tolerability profile (Mazighi et al., 2024).

### Protease activated receptors 4 (PAR4) inhibitors and other novel antiplatelet targets

There are several other antiplatelet drugs in early phases of development which show promise for the treatment of STEMI including the PAR4 receptor antagonists, GPIb-IX-V and Von Willebrand factor (VWF) blockers, 5-hydroxytryptamine receptor subtype 2A (5-HT2A), protein disulphide isomerase inhibitors, phosphoinositide 3-kinase β inhibitors and phosphodiesterase inhibitors (Alenazy and Thomas, 2021).

Exposed tissue factor at the site of vascular injury activates the coagulation system, resulting in thrombin generation and activation of PAR4 on the platelet surface. This causes further shape change of the platelets, release of granules and stimulates platelet aggregation. PAR4 also causes thrombus stabilisation, ADP release, activates P2Y12 and mediates platelet-leukocyte interaction (Alenazy and Thomas, 2021). In contrast to the PAR1, the main thrombin receptor, PAR4 is thought to have minimal involvement in haemostasis and is currently being investigated a potential antithrombotic agent. BMS-986120 and BMS p86141 are PAR4 antagonists that have been tested for their antithrombotic properties (Covic et al., 2000). BMS-986120 has been investigated in a phase I trial in which selectively blocking PAR4 resulted in reduced thrombus formation (Wilson et al., 2018). BMS-986141 was shown to prevent blood vessel occlusion by platelet in pre clinical models with minimal effects on haemostasis. Recently published results of the phase 1 randomised, double-blind, placebo-controlled study investigating the BMS-986141 showed it to be safe and well tolerated (Merali et al., 2023). Both of these PAR4 antagonists show a potential in the treatment of arterial thrombosis with a minimal effect on the bleeding (Priestley et al., 2022).

GPIb-IX-V is made up of four proteins GP1bα, GPIbβ, GPV and GPIX forming a complex that mediates initial platelet adhesion when cell injury occurs. Via the VWF domain, it forms a bridge between the GPIb-V-IX complex and the exposed collagen from the vascular sub-endothelium (Alenazy and Thomas, 2021). Caplacizumab, a nanobody that blocks VWF is currently being used for the treatments of adults with acquired thrombotic thrombocytopenic purpura (TTP) in preventing the development of potentially life threatening microvascular thrombosis. There is however an association of increased bleeding related events (Hollifield et al., 2020). Caplacizumab has been tested in a phase II study treating ACS patients but because it did not show a reduction in bleeding events in 30 days compared to abciximab, the trial was discontinued. There were major bleeding events reported when compared to a group treated with abciximab (Sinha, 2012; Duggan, 2018;).

5-hydroxytryptamine receptor subtype 2A (5-HT2A) is a G protein-coupled receptor stored in platelet dense granules. It is a weak platelet stimulus and is believed to enhance platelet activation and aggregation induced by major platelet ligands such as thrombin, ADP and epinephrine. Selective serotonin reuptake inhibitors (SSRIs) are used clinically as antidepressants. Long term use of SSRIs has been associated with a reduced risk of myocardial infarction (Alenazy and Thomas, 2021). A study that investigated the impact of the 5-Hydroxytrptamine receptor subtype 2A antagonist, sarpogrelate in addition to DAPT on acute coronary syndrome patients did not find a difference in ST resolution, TIMI flow or MACE. Left ventricular function did however improve in the triple therapy (treatment) group. No increase in bleeding was associated with sarpogrelate (Choi et al., 2017).

P-selectin functions as an adhesion molecule on the surface of stimulated platelets and epithelial cells. It is used as a marker of platelet activation and only expressed on the cell surface once a platelet is activated. P-selectin has a role in leukocyte-platelet interaction by binding to P-selectin glycoprotein ligand-1 which is present on leukocytes. It also stabilises platelet aggregates by facilitating platelet-platelet interaction (Alenazy and Thomas, 2021). Inclacumab, an anti-P-selectin monoclonal antibody has been investigated in the ACS setting and has been shown to reduce myocardial damage in patients with NSTEMI following PCI (Tardif et al., 2013; Choi et al., 2017). Inhibition of P-selectin has also shown improved microvascular flow in patients with sickle cell disease and prevention of vaso occlusive events. Crizanlizumab, a monoclonal antibody that inhibits P-selectin function has shown a reduction in sickle cell pain crisis. These findings have led to FDA approval of Crizanlizumab in sickle cell disease for the prevention of vaso occlusive events (Karki and Kutlar, 2021).

In summary, platelets play an important role in haemostasis and thrombus formation and are thought to play a key role in microvascular obstruction and reperfusion injury following STEMI. Since the ISIS2 trial publication, antiplatelet therapy has been a fundamental part of the pharmacological treatment of STEMI with strong evidence that newer more potent drugs acting on the P2Y_12_ receptor effectively reduce recurrent ischemic events and stent thrombosis. Evidence that these antiplatelet drugs can reduce microvascular obstruction, reperfusion injury and reduce infract size has however been inconclusive. Some of the novel antiplatelet drugs, such as GPVI inhibitors, discussed in this review have promise in the clinical setting of STEMI and require detailed investigation in both pre-clinical and clinical studies to document effects on efficacy and safety, particularly on microvascular obstruction, reperfusion injury and infarct size.
